# Xinmailong injection on left ventricular remodeling and inflammatory mediators in patients with CHF: a systematic review and meta-analysis

**DOI:** 10.3389/fphar.2024.1370448

**Published:** 2024-04-09

**Authors:** Xu Han, Xi Chen, Yanling Liu, Jie Yang, Wuzhi Nie, Mingjiu Yang, Xinglang Mou

**Affiliations:** ^1^ Chongqing Changshou Traditional Cinese Medicine Hospital, Chongqing, China; ^2^ Traditional Chinese Medicine Hospital Dianjiang Chongqing, Chongqing, China

**Keywords:** Xinmailong injection, chronic heart failure, left ventricular remodeling, inflammation mediators, randomized controlled trials, systematic review, meta-analysis

## Abstract

**Background:**

Chronic heart failure (CHF) is a prevalent and highly challenging cardiovascular disease associated with high mortality rates. The occurrence and progression of CHF are closely linked to left ventricular remodeling (LVR) and inflammation. Addressing LVR and reducing inflammation can significantly slow down the progression of CHF and improve patient prognosis.

**Objective:**

To evaluate the effects of Xinmailong injection (XMLI) on LVR and inflammatory mediators in CHF patients.

**Method:**

The randomized controlled trials investigating the effectiveness of XMLI treatment for CHF were retrieved from eight databases up until 31 December 2023. To evaluate the methodological quality of included studies, the Cochrane bias risk tool was employed. Furthermore, statistical analysis, sensitivity analysis, and publication bias assessment were conducted using Stata 17.0 software.

**Result:**

Compared with conventional treatment (CT), the combination therapy of XMLI and CT significantly improved LVR and reduced inflammatory mediators, mainly manifested by an increase in LVEF (MD = 6.40, 95% CI: 5.25 to 7.55, *p* = 0.000), a decrease in LVEDD (MD = −4.63, 95% CI: −5.69 to −3.57, *p* = 0.000) and LVESD (MD = −4.00, 95% CI: −5.50 to −2.50, *p* = 0.000), as well as a decrease in TNF-α (MD = −7.93, 95% CI: −9.86 to −6.00, *p* = 0.000), IL-6 (MD = −5.25, 95% CI: −6.59 to −3.92, *p* = 0.000), IL-18 (MD = −36.07, 95% CI: −46.76 to −25.38, *p* = 0.000), CRP (MD = −4.41, 95% CI: −6.40 to −2.42, *p* = 0.000), hs-CRP (MD = −4.90, 95% CI: −5.71 to −4.08, *p* = 0.000), and an increase in IL-10 (MD = 20.19, 95% CI: 10.42 to 29.97, *p* = 0.000). In addition, the combination therapy showed enhanced clinical efficacy (OR = 4.08, 95% CI: 3.10 to 5.37, *p* = 0.000), decreased expression levels of BNP (MD = −138.48, 95% CI: −155.48 to −121.48, *p* = 0.000), and NT-pro BNP (MD = −315.63, 95% CI: −359.25 to −272.00, *p* = 0.000), and increased the 6-MWD (MD = 71.02, 95% CI: 57.23 to 84.81, *p* = 0.000). It is noteworthy that the combination therapy did not lead to an increase in the incidence of adverse reactions (OR = 1.01, 95% CI: 0.68 to 1.50, *p* = 0.97).

**Conclusion:**

This systematic review and meta-analysis demonstrated the superiority of combining XMLI and CT therapies over CT alone in improving LVR and reducing inflammatory mediators in patients with CHF. Importantly, this combination therapy does not increase adverse reactions. However, it is crucial to exercise caution while interpreting the survey results due to the limited quality of the included studies.

**Systematic Review Registration:** https://www.crd.york.ac.uk/PROSPERO/display_record.php?RecordID=492715, Identifier CRD42023492715.

## 1 Introduction

Chronic heart failure (CHF) is a major public health problem, affecting 26 million people worldwide and leading to a high incidence rate and mortality. This condition brings a huge burden to both patients and society due to its complex clinical syndrome caused by multiple etiologies (Conrad et al., 2018; Mensah et al., 2023). CHF can be classified into two distinct subtypes, namely, heart failure with reduced ejection fraction (HFrEF, LVEF less than 40%) and heart failure with mid-range ejection fraction (HFmrEF, LVEF ranging from 40% to 49%). While the pathological and physiological mechanisms of CHF are not fully understood, left ventricular remodeling (LVR) and increased inflammation are known characteristics of the condition (Dick and Epelman, 2016; Aimo et al., 2019). There is a close relationship between LVR, inflammatory response, and the occurrence and progression of CHF (Hartupee and Mann, 2013; Dick and Epelman, 2016; Tong et al., 2018). Elevated levels of pro-inflammatory factors have been found to be positively correlated with the severity and adverse outcomes of CHF (Smart and Steele, 2011). Therefore, an important strategy to alleviate symptoms and improve prognosis in CHF patients is to enhance LVR and reduce inflammation.

LVR, which refers to the structural and functional changes in the left ventricle of the heart, is a consequence of various etiologies that contribute to the development of CHF (Aimo et al., 2019). These changes in ventricular structure and function can significantly impair cardiac performance, leading to worsened symptoms and outcomes for patients. Numerous studies have demonstrated the potential of interventions aimed at improving LVR to delay or even reverse the progression of CHF (Biering-Sørensen et al., 2020; Lee et al., 2021). In addition to LVR, inflammation has been recognized as a key pathophysiological factor in CHF (Hartupee and Mann, 2013). Elevated levels of pro-inflammatory factors, including tumor necrosis factor α (TNF-a), interleukin-6 (IL-6), IL-18, C-reactive protein (CRP), and hypersensitive C-reactive protein (hs-CRP), have been closely associated with the severity and adverse consequences of the disease in CHF patients (Arvunescu et al., 2023). Promising results have been reported in targeting or regulating the activity of these inflammatory mediators (Murphy et al., 2020). Consequently, targeting both LVR and inflammatory mediators has emerged as a significant therapeutic strategy for alleviating symptoms and improving prognosis in patients with CHF.

Xinmailong injection (XMLI) is a composite peptide injection extracted from *Periplaneta americana* L, containing adenosine, inosine, protocatechuic acid, and pyroglutamyl dipeptide as its main active ingredients (Qi et al., 2017). Modern pharmacological studies have elucidated the cardioprotective properties of XMLI, notably in inhibiting oxidative stress (Jiang et al., 2021) and inflammatory response (Jin et al., 2022), regulating cell autophagy (Li et al., 2016), and modulating cytokine expression (Liu et al., 2017). Jiang et al. (Jiang et al., 2021) observed that XMLI modulates HO-1 mediated lysosomal function and autophagy in H9C2 cells, reduces oxidative stress and mitigates DOX-induced cardiac toxicity. Jin et al. (Jin et al., 2022) revealed that XMLI can reduce ROS production, minimize inflammatory response, and decrease cell apoptosis by improving PKC and PLA2 protein-mediated myocardial ischemia. Li et al. (Li et al., 2016) demonstrated that XMLI targets autophagy by activating the PI3K/Akt pathway and inhibiting Erk1/2 and P38 MAPK pathways, effectively alleviating epirubicin-induced cardiomyopathy. Additionally, Liu et al. (Liu et al., 2017) highlighted that XMLI inhibits connective tissue growth factor (CTGF), enhancement of heart function, and reduction of alcoholic myocardial fibrosis in rat models. As a result, XMLI is widely utilized as an adjuvant medication for CHF in China. However, there is a limited comprehensive evaluation of XMLI’s impact on LVR and inflammatory mediators in patients with CHF. Given the crucial role of LVR and inflammation in the development and progression of CHF, this study aims to bridge this knowledge gap through a meta-analysis of clinical randomized controlled trials (RCTs).

## 2 Methods

### 2.1 Study registration

This meta-analysis followed the PRISMA (preferred reporting item for systematic evaluation and meta-analysis) guidelines (Hutton et al., 2015) and was registered with PROSPERO (NO. CRD42023492715).

### 2.2 Database and search strategy

To investigate the treatment of CHF with XMLI, the two reviewers conducted an extensive literature search. Relevant studies were searched and retrieved from various databases, including PubMed, Embase, Web of Science, Cochrane Library, Wanfang Data, China Knowledge Infrastructure Database (CNKI), China Biomedical Database (CBM), and China Science and Technology Journal Database (VIP). The search employed a combination of MeSH terminology and textual terminology. The search terms include “Xinmailong injection”, “Xinmailong ", “heart failure”, and “chronic heart failure”. The search was conducted from their establishment to 31 December 2023. In addition, the reviewers manually searched the reference lists of published literature to ensure comprehensive coverage. Detailed search strategies can be found in the Supplementary Material.

### 2.3 Inclusion and exclusion criteria

According to the PICOS principle, the following conditions must be met for inclusion in the study: 1) RCTs without any language restrictions on publication. 2) Participants diagnosed with CHF, aged 18 and above. 3) The intervention group received a combination of XMLI and conventional treatment (CT), while the control group received CT based on heart failure (HF) guidelines. 4) The primary outcome measures primarily focus on LVR (LVEF, LVEDD, LVESD) and inflammatory mediators (TNF-α, IL-6, IL-10, IL-18, CRP, hs-CRP).

The exclusion criteria are as follows: 1) Non-RCTs. 2) Unstable heart failure. 3) Repeated publication, retaining only complete data for research. 4) Research without primary outcome measures. 5) The full study cannot be obtained through databases or other means.

### 2.4 Data extraction

Two reviewers (XH and XC) independently evaluated the included studies and extracted data. If any discrepancies or disagreements arose during the evaluation process, a third reviewer (MY) was available for discussion and resolution. The data extraction was conducted by the two researchers (XH and XC) using a pre-established table that included several important parameters. These parameters encompassed the article title, first author, publication year, sample size, intervention drugs, dosage and course of treatment, outcome measures, and adverse reactions.

### 2.5 Quality assessment

Two reviewers (YL and JY) independently evaluated the methodological quality of the included studies using the Cochrane Collaboration risk of bias tool (Sterne et al., 2019). The evaluation encompassed various aspects, including randomization methods, allocation concealment, blinding, completeness of outcome data, selective reporting, and other sources of bias. The results of the evaluations were then cross-checked to ensure accuracy and consistency. The risk of bias for each study was classified as low, unclear, or high. Any disagreements that arose during the methodological quality assessment process were resolved through discussions involving third reviewer (XM).

### 2.6 Data analysis

All meta-analyses were conducted using RevMan5.4 and Stata 17.0 software. For dichotomous data, a 95% confidence interval (CI) risk ratio (RR) was calculated, while continuous data utilized a mean difference (MD) with a 95% CI. Heterogeneity among the included studies was evaluated using I^2^. I^2^ ≤ 50% was considered as low heterogeneity, and a fixed-effects model was applied. Conversely, a random-effects model was applied. Furthermore, subgroup analysis was performed based on differences in LVEF to investigate possible factors influencing the results. Sensitivity analysis was performed on the primary outcome measures to evaluate the impact of individual studies on the combined effect size. The Egge’s tests were employed to test for potential publication bias.

## 3 Results

### 3.1 Search results and study characteristics

**FIGURE 1:**
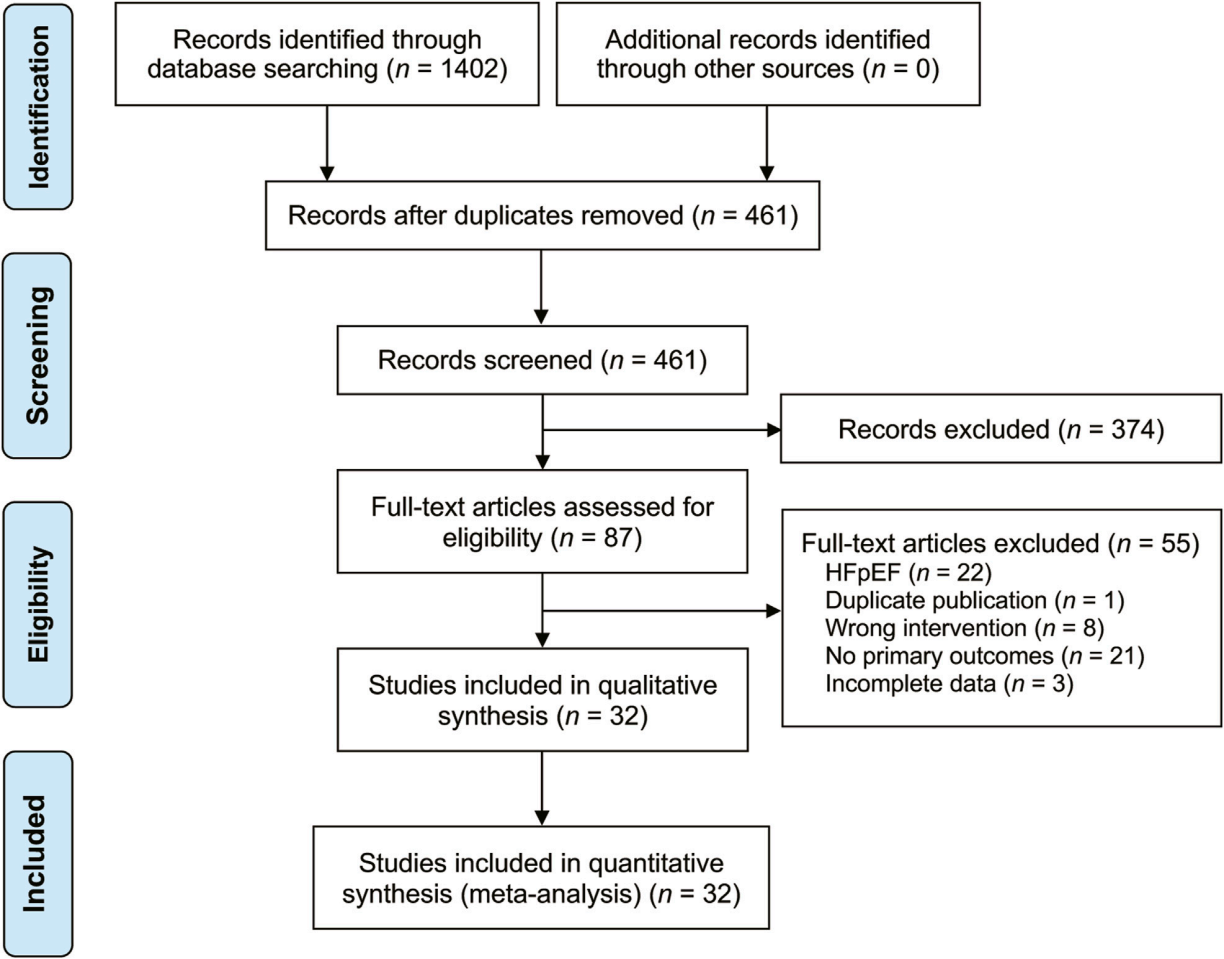
The PRISMA study flowchart of study search.

A total of 1,402 related studies were retrieved through a systematic search. After deduplication and screening, 32 studies (Bao, 2017; Chen, 2012; Guo et al., 2016; Han, 2019; Huang and Cheng, 2022; Hui and Wu, 2017; Li et al., 2022; Li et al., 2016; Li et al., 2020; Liu et al., 2015; Mao, 2019; Song, 2022; Song et al., 2016; Su, 2020; Wang, 2019; Wei, 2023; Wu et al., 2017; Xi et al., 2019; Xu et al., 2018; Xu and Cao, 2019; Yan et al., 2019; Yao and Yang, 2022; Ye et al., 2017; Yu and Zhang, 2023; Yuan, 2019; Zhang et al., 2021; Zhang, 2020; Zhang et al., 2013; Zhao et al., 2019; Zhao, 2019; Zhou and Xu, 2020; Zhu et al., 2017) published from 2012 to 2023 were selected for the final analysis. The literature search results are displayed in Figure 1. These studies were conducted in China and involved 3,346 patients (1855 males and 1,491 females) with varying sample sizes (ranging from 23 to 175) and treatment courses (ranging from 5 to 28 days). The control group received CT recommended by the HF guidelines, while the treatment group received XMLI combined with CT. No statistically significant differences in general information were found between the two groups. The included studies provided results on various parameters, including LVEF (Chen, 2012; Zhang et al., 2013; Liu et al., 2015; Guo et al., 2016; Li et al., 2016; Song et al., 2016; Bao, 2017; Hui and Wu, 2017; Wu et al., 2017; Ye et al., 2017; Zhu et al., 2017; Xu et al., 2018; Han, 2019; Mao, 2019; Song, 2022; Wang, 2019; Xi et al., 2019; Xu and Cao, 2019; Yan et al., 2019; Yuan, 2019; Zhao, 2019; Zhao et al., 2019; Li et al., 2020; Su, 2020; Zhang, 2020; Zhou and Xu, 2020; Zhang et al., 2021; Huang and Cheng, 2022; Li et al., 2022; Yao and Yang, 2022; Wei, 2023; Yu and Zhang, 2023), LVEDD (Chen, 2012; Liu et al., 2015; Hui and Wu, 2017; Ye et al., 2017; Zhu et al., 2017; Han, 2019; Mao, 2019; Song, 2022; Xi et al., 2019; Zhao, 2019; Zhao et al., 2019; Li et al., 2020; Su, 2020; Zhou and Xu, 2020; Zhang et al., 2021; Huang and Cheng, 2022; Li et al., 2022; Wei, 2023; Yu and Zhang, 2023), LVESD (Liu et al., 2015; Song, 2022; Ye et al., 2017; Zhu et al., 2017; Zhao, 2019; Huang and Cheng, 2022; Li et al., 2022; Wei, 2023; Yu and Zhang, 2023), TNF-α (Zhang et al., 2013; Ye et al., 2017; Zhu et al., 2017; Yuan, 2019; Zhao et al., 2019; Huang and Cheng, 2022; Song, 2022; Yao and Yang, 2022; Wei, 2023), IL-6 (Zhang et al., 2013; Li et al., 2016; Song, 2022; Bao, 2017; Ye et al., 2017; Zhu et al., 2017; Yuan, 2019; Zhao et al., 2019; Huang and Cheng, 2022; Yao and Yang, 2022; Wei, 2023), IL-10 (Guo et al., 2016; Han, 2019; Zhao et al., 2019; Zhou and Xu, 2020; Wei, 2023), IL-18 (Guo et al., 2016; Han, 2019; Zhao et al., 2019; Zhou and Xu, 2020; Wei, 2023), CRP (Chen, 2012; Zhang et al., 2013; Hui and Wu, 2017; Ye et al., 2017; Zhu et al., 2017; Xu et al., 2018; Zhao, 2019), hs-CRP (Li et al., 2016; Song et al., 2016; Bao, 2017; Wu et al., 2017; Mao, 2019; Xi et al., 2019; Xu and Cao, 2019; Yan et al., 2019; Zhao, 2019; Zhao et al., 2019; Li et al., 2020; Zhang, 2020; Zhou and Xu, 2020; Zhang et al., 2021; Yu and Zhang, 2023), clinical efficacy (Chen, 2012; Li et al., 2016; Song et al., 2016; Bao, 2017; Hui and Wu, 2017; Ye et al., 2017; Zhu et al., 2017; Xu et al., 2018; Wang, 2019; Xi et al., 2019; Xu and Cao, 2019; Yuan, 2019; Zhao, 2019; Zhao et al., 2019; Li et al., 2020; Su, 2020; Zhang, 2020; Zhou and Xu, 2020; Zhang et al., 2021; Li et al., 2022; Yu and Zhang, 2023), 6-MWD (Zhang et al., 2013; Li et al., 2016; Bao, 2017; Hui and Wu, 2017; Wu et al., 2017; Xu et al., 2018; Mao, 2019; Wang, 2019; Wei, 2023), BNP (Liu et al., 2015; Song, 2022; Guo et al., 2016; Li et al., 2016; Song et al., 2016; Wu et al., 2017; Zhu et al., 2017; Xu et al., 2018; Wang, 2019; Yan et al., 2019; Zhao, 2019; Su, 2020; Zhang et al., 2021; Li et al., 2022; Yao and Yang, 2022), and NT-pro BNP (Bao, 2017; Hui and Wu, 2017; Ye et al., 2017; Mao, 2019; Xi et al., 2019; Xu and Cao, 2019; Li et al., 2020; Zhang, 2020; Zhou and Xu, 2020; Huang and Cheng, 2022; Wei, 2023; Yu and Zhang, 2023). Among these studies, only 10 studies (Ye et al., 2017; Zhu et al., 2017; Xu et al., 2018; Xi et al., 2019; Li et al., 2020; Su, 2020; Zhang et al., 2021; Huang and Cheng, 2022; Li et al., 2022; Yao and Yang, 2022) reported adverse reactions. The basic characteristics of the included studies are present in Table 1.

**TABLE 1:** Study characteristics.

Included studies	Sample size		Mean age (years)		Sex (M/F)		Heart failure subtypes	Interventions		Treatment duration	CT drugs	Outcomes
	T	C	T	C	T	C		T	C			
Bao (2017)	60	60	56.7	56.6	32/28	33/27	HFrEF	Xinmailong injection, 6mL, bid	CT	14d	ACEI, diuretic, nitrates, β-receptor blockers, aspirin, clopidogrel, statins	①⑤⑨⑩⑪⑬
Chen (2012)	53	47	69.3 ± 6.9		43/57		HFrEF	Xinmailong injection, 5 mg/kg, bid	CT	5d	ACEI, diuretic	①②⑧⑩
Guo et al. (2016)	34	34	73.61 ± 8.46		39/29		HFmrEF	Xinmailong injection, 4mL, bid	CT	10d	Digoxin, ACEI, diuretic, β-receptor blockers	①⑥⑦⑫
Han (2019)	63	63	71.09 ± 3.43	70.88 ± 3.37	36/27	34/29	HFmrEF	Xinmailong injection, 4mL, bid	CT	10d	Digoxin, diuretic, spironolactone, β-receptor blockers	①②⑥⑦
Huang and Cheng (2022)	63	63	73.63 ± 5.75	73.14 ± 5.69	36/27	39/24	HFmrEF	Xinmailong injection, 5 mg/kg, qd	CT	28d	Digoxin, ACEI, diuretic, spironolactone	①②③④⑤⑬⑭
Hui and Wu (2017)	60	60	73.4 ± 5.7	72.6 ± 5.8	32/28	34/26	HFmrEF	Xinmailong injection, 5 mg/kg, bid	CT	15d	Digoxin, ACEI, diuretic, nitrates, β-receptor blockers	①②⑧⑩⑪⑬
Li et al. (2022)	175	175	59.64 ± 6.12	60.15 ± 6.03	70/105	75/100	HFmrEF	Xinmailong injection, 5 mg/kg, bid	CT	10d	ACEI, diuretic, nitrates, β-receptor blockers, aspirin, statins	①②③⑩⑫⑭
Li et al. (2016)	23	23	56.87 ± 5.43		28/18		HFrEF	Xinmailong injection, 6mL, bid	CT	14d	ACEI, β-receptor blockers, aspirin, clopidogrel, statins	①⑤⑨⑩⑪⑫
Li et al. (2020)	60	60	65.53 ± 2.72	65.94 ± 2.96	32/28	36/24	HFrEF	Xinmailong injection, 5 mg/kg, bid	CT	14d	ACEI, diuretic, β-receptor blockers	①②⑨⑩⑬⑭
Liu et al. (2015)	60	76	44.2 ± 3.7	45.2 ± 3.5	43/17	48/28	HFmrEF	Xinmailong injection, 4mL, bid	CT	14d	ACEI, diuretic, β-receptor blockers	①②③⑫
Mao (2019)	26	26	46.87 ± 5.41		38/14		HFrEF	Xinmailong injection, 5 mg/kg, bid	CT	10d	ACEI, diuretic, nitrates, β-receptor blockers	①②⑨⑪⑬
Song (2022)	60	60	69.1 ± 4.0	68.3 ± 4.3	38/22	35/25	HFmrEF	Xinmailong injection, 6mL, qd	CT	7d	ACEI, diuretic, nitrates, β-receptor blockers, aspirin, statins	①②③④⑤⑫
Song et al. (2016)	42	42	59.65 ± 4.46	59.63 ± 4.43	20/22	21/21	HFrEF	Xinmailong injection, 5 mg/kg, bid	CT	10d	Digoxin, diuretic, nitrates, β-receptor blockers	①⑨⑩⑫
Su (2020)	51	51	48.5 ± 4.1	47.8 ± 4.3	23/28	25/26	HFmrEF	Xinmailong injection, 5 mg/kg, bid	CT	10d	Digoxin, diuretic, nitrates, β-receptor blockers, statins	①②⑩⑫⑭
Wang (2019)	58	58	59.54 ± 7.80	59.39 ± 7.92	27/31	28/30	HFmrEF	Xinmailong injection, 5 mg/kg, bid	CT	14d	Digoxin, ACEI, diuretic, nitrates, β-receptor blockers	①⑩⑪⑫
Wei (2023)	42	42	44.42 ± 3.23	43.57 ± 3.03	25/17	27/15	HFrEF	Xinmailong injection, 6mL, qd	CT	10d	ACEI, diuretic, nitrates, β-receptor blockers, aspirin, clopidogrel, statins	①②③④⑤⑥⑦⑪⑬
Wu et al. (2017)	48	42	54.05 ± 3.96	56.13 ± 4.87	28/20	31/11	HFmrEF	Xinmailong injection, 5 mg/kg, bid	CT	10d	Digoxin, ACEI, diuretic, nitrates, β-receptor blockers	①⑨⑪⑫
Xi et al. (2019)	30	30	58.46 ± 4.39	58.83 ± 4.10	18/12	21/9	HFmrEF	Xinmailong injection, 4mL, bid	CT	14d	ACEI, diuretic, spironolactone	①②⑨⑩⑬⑭
Xu et al. (2018)	44	44	66.1 ± 12.3	65.3 ± 11.6	24/20	19/25	HFrEF	Xinmailong injection, 5 mg/kg, bid	CT	7d	ACEI, diuretic, nitrates, β-receptor blockers, aspirin, clopidogrel, statins	①⑧⑩⑪⑫⑭
Xu and Cao (2019)	57	51	61.39 ± 5.73	60.28 ± 6.41	25/32	24/27	HFrEF	Xinmailong injection, 5 mg/kg, bid	CT	14d	ACEI, diuretic, nitrates	①⑨⑩⑬
Yan et al. (2019)	61	61	87.12 ± 6.98	86.35 ± 7.67	37/24	39/22	HFrEF	Xinmailong injection, 6mL, bid	CT	10d	ACEI, diuretic, aspirin, statins	①⑨⑫
Yao and Yang (2022)	46	46	62.13 ± 8.71	61.23 ± 8.69	26/20	27/19	HFrEF	Xinmailong injection, 5 mg/kg, bid	CT	5d	ACEI, diuretic, spironolactone	①④⑤⑫⑭
Ye et al. (2017)	63	63	71.31 ± 11.36	74.01 ± 13.22	39/24	43/20	HFrEF	Xinmailong injection, 5 mg/kg, bid	CT	14d	ACEI, diuretic, β-receptor blockers, aspirin, clopidogrel, statins	①②③④⑤⑧⑩⑬⑭
Yu and Zhang (2022)	45	45	48.19 ± 13.78	48.27 ± 14.46	25/20	23/22	HFrEF	Xinmailong injection, 5 mg/kg, bid	CT	10d	ACEI, diuretic, β-receptor blockers	①②③⑨⑩⑬
Yuan (2019)	43	43	67.85 ± 8.97	68.02 ± 9.35	27/16	29/14	HFrEF	Xinmailong injection, 6mL, qd	CT	7d	ACEI, diuretic, β-receptor blockers, aspirin, clopidogrel, statins	①④⑤⑩
Zhang et al. (2021)	65	65	68.10 ± 4.25	67.85 ± 4.11	40/25	45/20	HFmrEF	Xinmailong injection, 5 mg/kg, bid	CT	10d	Digoxin, diuretic, spironolactone, β-receptor blockers, statins	①②⑨⑩⑫⑭
Zhang (2020)	40	40	63.3 ± 2.3	64.8 ± 3.5	21/19	19/21	HFrEF	Xinmailong injection, 4mL, bid	CT	7d	ACEI, nitrates, statins	①⑨⑩⑬
Zhang et al. (2013)	30	30	57.3 ± 7.4		19/11	21/9	HFrEF	Xinmailong injection, 6mL, bid	CT	10d	ACEI, diuretic, nitrates, β-receptor blockers, aspirin, clopidogrel, statins	①④⑤⑧⑪
Zhao et al. (2019)	30	30	59.6 ± 11.3		12/18	15/15	HFrEF	Xinmailong injection, 5 mg/kg, bid	CT	10d	Digoxin, diuretic, β-receptor blockers	①②③④⑤⑥⑦⑧⑩
Zhao (2019)	49	49	63.58 ± 3.84	64.15 ± 3.67	28/21	29/20	HFrEF	Xinmailong injection, 4mL, bid	CT	14d	ACEI, diuretic, β-receptor blockers	①②⑨⑩
Zhou and Xu (2020)	50	50	66.57 ± 3.16	65.66 ± 3.31	27/23	29/21	HFmrEF	Xinmailong injection, 5 mg/kg, bid	CT	14d	Digoxin, ACEI, diuretic, nitrates, β-receptor blockers	①②⑥⑦⑨⑩⑬
Zhu et al. (2017)	43	43	67.29 ± 5.19	68.14 ± 5.24	25/18	23/20	HFmrEF	Xinmailong injection, 5 mg/kg, bid	CT	14d	Digoxin, ACEI, diuretic, aspirin	①②③④⑤⑧⑩⑫⑭

C, control group; T, treatment group; M, male; F, female; d, days; qd, quaque in die; bid, bis in die; CT: conventional treatment; ACEI: angiotensin-converting enzyme inhibitor. HFrEF: heart failure with reduced ejection fraction; HFmrEF: heart failure with mid-range ejection fraction. Outcomes: ①LVEF; ②LVEDD; ③LVESD; ④TNF-α; ⑤IL-6; ⑥IL-10; ⑦IL-18; ⑧CRP; ⑨hs-CRP; ⑩Clinical efficacy; ⑪6-MWD; ⑫BNP; ⑬NT-pro BNP; ⑭Adverse reactions.

### 3.2 Risk of bias assessment

**FIGURE 2:**
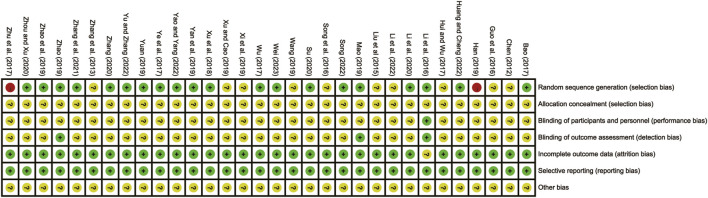
Bias risk assessment of included studies.

The overall quality of the included studies varied. 20 studies (Bao, 2017; Huang and Cheng, 2022; T. T; Li et al., 2016; Li et al., 2020; Mao, 2019; Song, 2022; Su, 2020; Wei, 2023; Wu et al., 2017; Xu et al., 2018; Yan et al., 2019; Yao and Yang, 2022; Ye et al., 2017; Yu and Zhang, 2023; Yuan, 2019; Zhang et al., 2021; Zhang, 2020; Zhao et al., 2019; Zhao, 2019; Zhou and Xu, 2020) utilized the low-risk random number table method. Conversely, 10 studies (Chen, 2012; Zhang et al., 2013; Liu et al., 2015; Guo et al., 2016; Song et al., 2016; Hui and Wu, 2017; Wang, 2019; Xi et al., 2019; Xu and Cao, 2019; Li et al., 2022) lacked clear descriptions of randomization, resulting in an unclear risk assessment. Two studies (Zhu et al., 2017; Han, 2019) were considered high-risk as they grouped patients based on admission order. None of the studies reported hidden allocation, leading to an unclear risk assessment for all of them. In terms of design, four studies (T. T. Li et al., 2016; Mao, 2019; Yan et al., 2019; Zhao, 2019) were multicenter double-blind tests, which were considered to be low-risk. Additionally, all included studies were published during a period of low risk of selective reporting and were given priority based on their locality. However, none of the studies clearly indicated the presence of other biases, resulting in an overall unclear risk assessment. The risk of bias assessment is detailed in Figure 2.

### 3.3 LVR parameters

#### 3.3.1 LVEF

**FIGURE 3:**
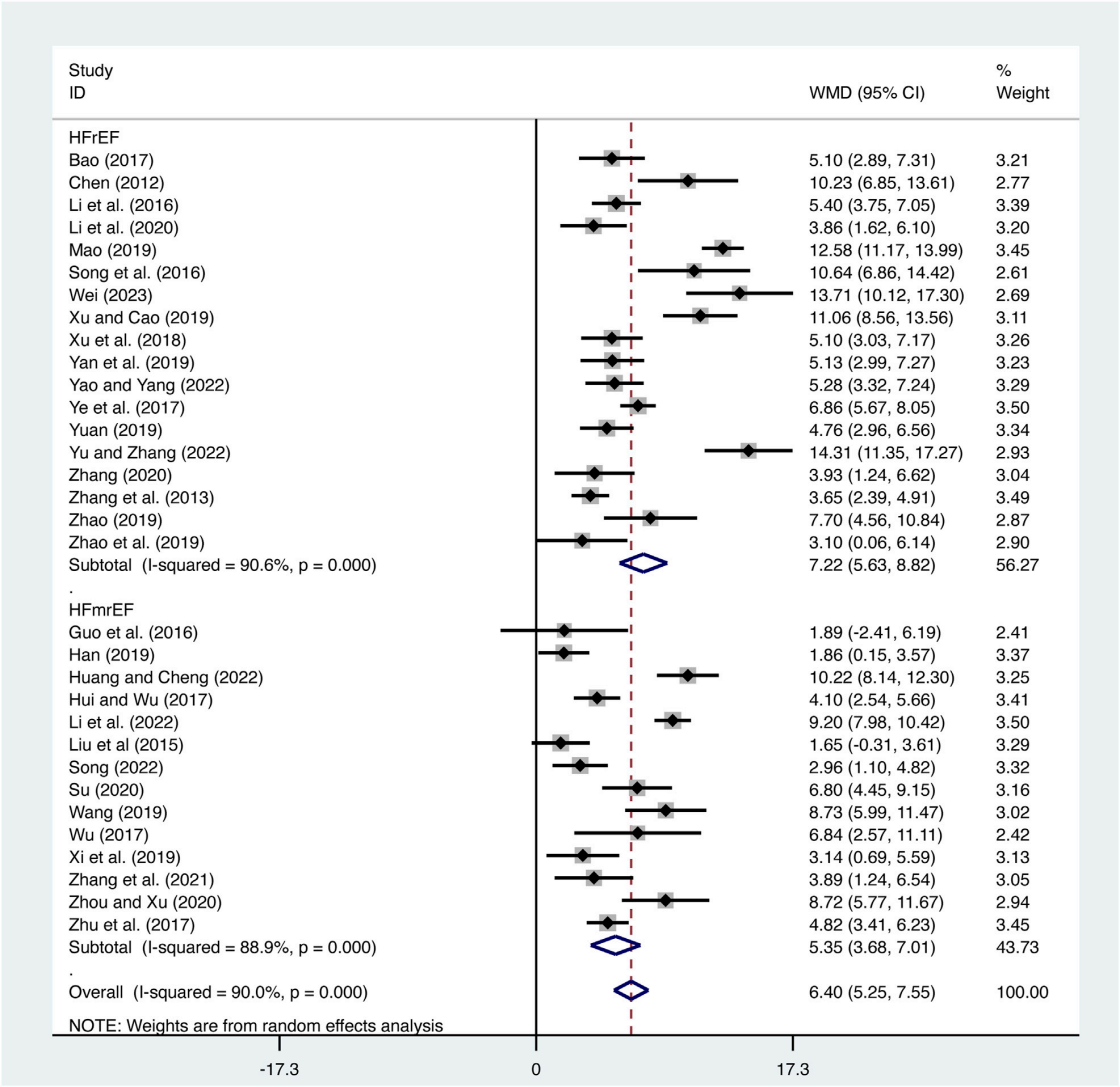
Forest plot for LVEF.

32 studies (Bao, 2017; Chen, 2012; Guo et al., 2016; Han, 2019; Huang and Cheng, 2022; Hui and Wu, 2017; Li et al., 2022; T. T; Li et al., 2016; Li et al., 2020; Liu et al., 2015; Mao, 2019; Song, 2022; Song et al., 2016; Su, 2020; Wang, 2019; Wei, 2023; Wu et al., 2017; Xi et al., 2019; Xu et al., 2018; Xu and Cao, 2019; Yan et al., 2019; Yao and Yang, 2022; Ye et al., 2017; Yu and Zhang, 2023; Yuan, 2019; Zhang et al., 2021; Zhang, 2020; Zhang et al., 2013; Zhao et al., 2019; Zhao, 2019; Zhou and Xu, 2020; Zhu et al., 2017) evaluated LVEF with high heterogeneity (*I* ^*2*^ = 90.0%, *p* = 0.000) and merged it with a random-effects model. The combination therapy of XMLI and CT significantly improved LVEF compared to CT alone (MD = 6.40, 95% CI: 5.25 to 7.55, *p* = 0.000, Figure 3). Subgroup analysis based on different subtypes of HF revealed a noteworthy enhancement in LVEF for patients with HFrEF (MD = 7.22, 95% CI: 5.63 to 8.82, *p* = 0.000, Figure 3) and HFmrEF (MD = 5.35, 95% CI: 3.68 to 7.01, *p* = 0.000, Figure 3) when the combination therapy was administered. Interestingly, the improvement was particularly prominent among patients with HFrEF.

#### 3.3.2 LVEDD

**FIGURE 4:**
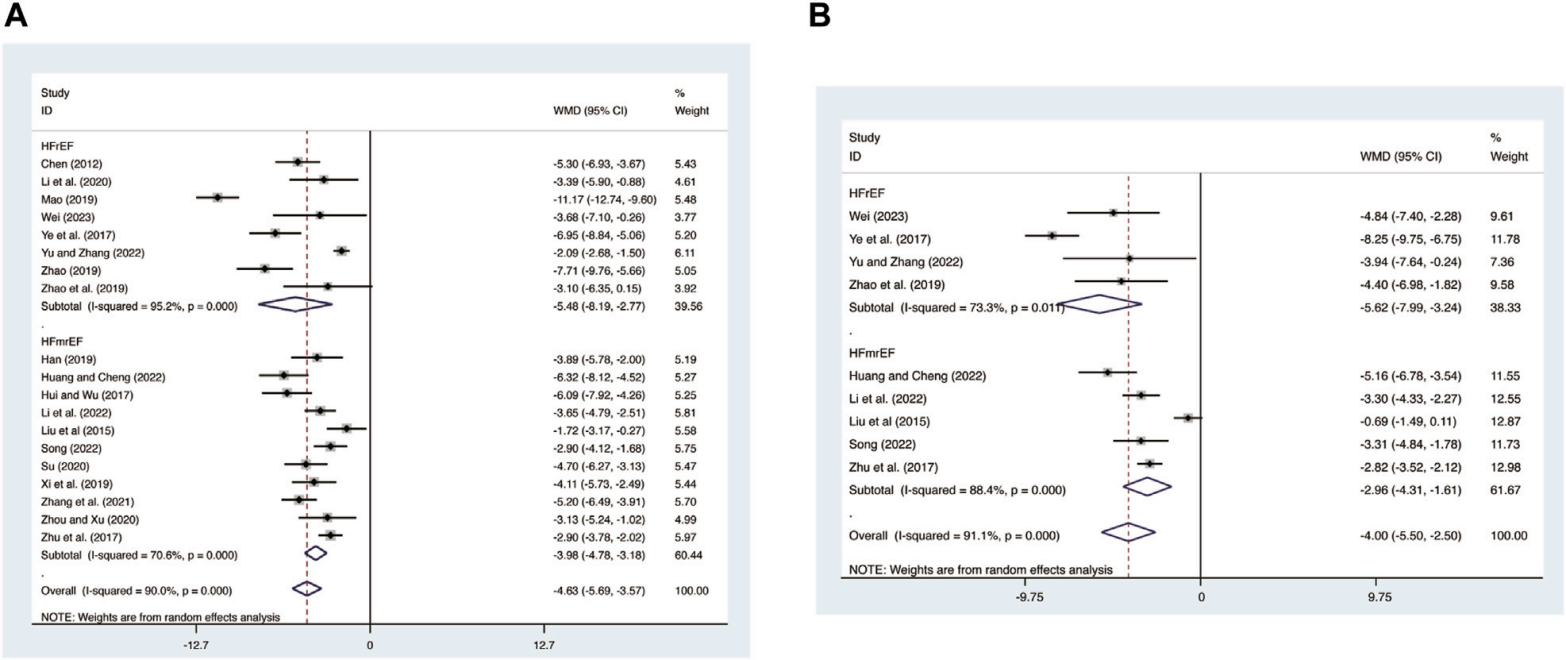
Forest plot for LVEDD and LVESD. **(A)** LVEDD. **(B)** LVESD.

19 studies (Chen, 2012; Liu et al., 2015; Hui and Wu, 2017; Ye et al., 2017; Zhu et al., 2017; Han, 2019; Mao, 2019; Song, 2022; Xi et al., 2019; Zhao, 2019; Zhao et al., 2019; Li et al., 2020; Su, 2020; Zhou and Xu, 2020; Zhang et al., 2021; Huang and Cheng, 2022; Li et al., 2022; Wei, 2023; Yu and Zhang, 2023) evaluated LVEDD with high heterogeneity (*I* ^*2*^ = 90.0%, *p* = 0.000) and merged it with a random-effects model. The combination therapy of XMLI and CT significantly reduced LVEDD compared to CT alone (MD = −4.63, 95% CI: −5.69 to −3.57, *p* = 0.000, Figure 4A). Subgroup analysis based on different subtypes of HF revealed a noteworthy reduction in LVEDD for patients with HFrEF (MD = −5.48, 95% CI: −8.19 to −2.77, *p* = 0.000, Figure 4A) and HFmrEF (MD = −3.98, 95% CI: −4.78 to −3.18, *p* = 0.000, Figure 4A) when the combination therapy was administered. Interestingly, the reduction was particularly prominent among patients with HFrEF.

#### 3.3.3 LVESD

Nine studies (Liu et al., 2015; Song, 2022; Ye et al., 2017; Zhu et al., 2017; Zhao, 2019; Huang and Cheng, 2022; Li et al., 2022; Wei, 2023; Yu and Zhang, 2023) evaluated LVESD with high heterogeneity (*I* ^*2*^ = 91.1%, *p* = 0.000) and merged it with a random-effects model. The combination therapy of XMLI and CT significantly reduced LVESD compared to CT alone (MD = −4.00, 95% CI: −5.50 to −2.50, *p* = 0.000, Figure 4B). Subgroup analysis based on different subtypes of HF revealed a noteworthy reduction in LVESD for patients with HFrEF (MD = −5.62, 95% CI: −7.99 to −3.24, *p* = 0.000, Figure 4B) and HFmrEF (MD = −2.96, 95% CI: −4.31 to −1.61, *p* = 0.000, Figure 4B) when the combination therapy was administered. Interestingly, the reduction was particularly prominent among patients with HFrEF.

### 3.4 Inflammatory mediators

#### 3.4.1 TNF-α

**FIGURE 5:**
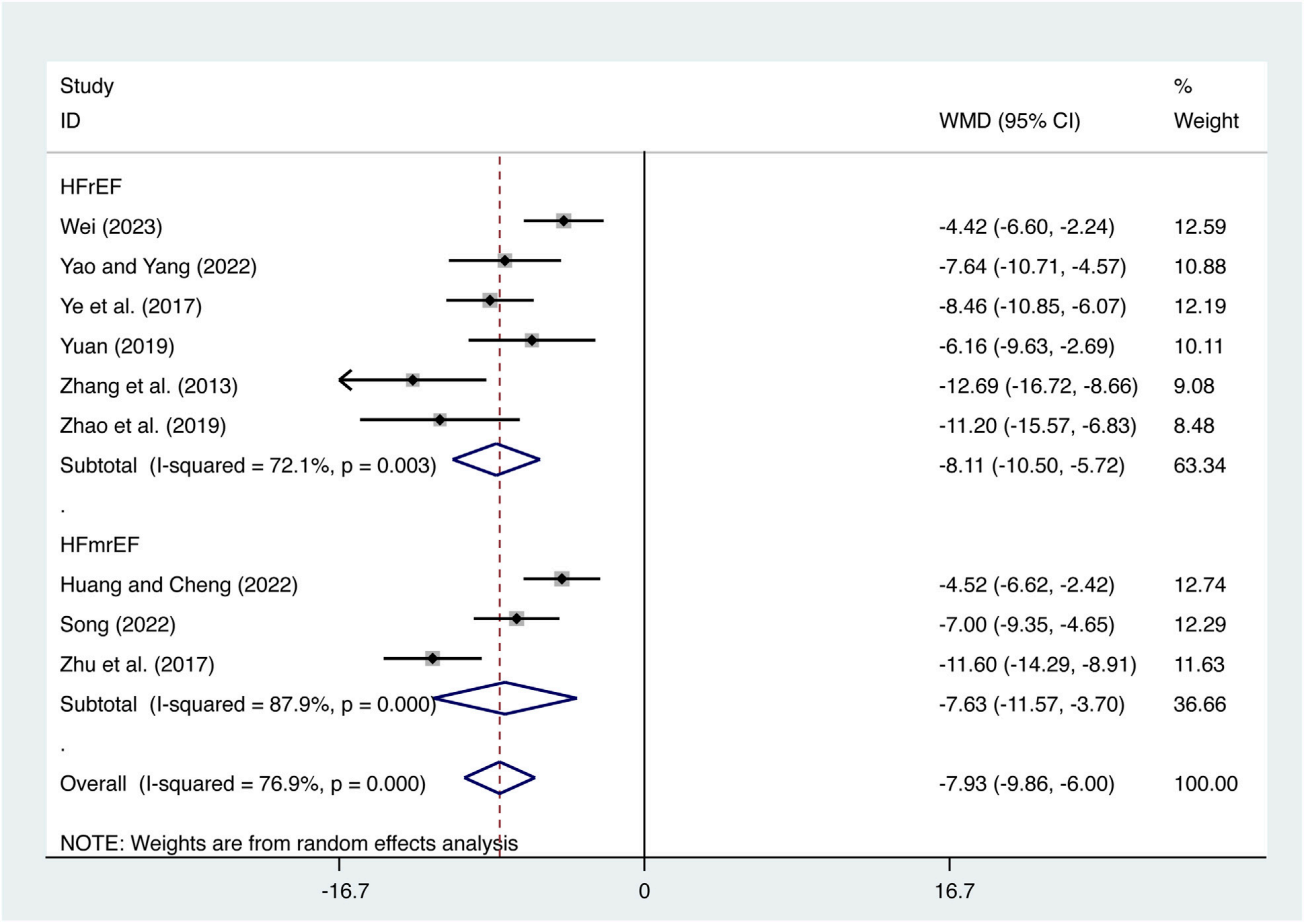
Forest plot for TNF-α expression levels.

Nine studies (Zhang et al., 2013; Ye et al., 2017; Zhu et al., 2017; Yuan, 2019; Zhao et al., 2019; Huang and Cheng, 2022; Song, 2022; Yao and Yang, 2022; Wei, 2023) evaluated TNF-α expression levels with high heterogeneity (*I* ^*2*^ = 76.9%, *p* = 0.000) and merged it with a random-effects model. The combination therapy of XMLI and CT significantly reduced TNF-α expression levels compared to CT alone (MD = −7.93, 95% CI: −9.86 to −6.00, *p* = 0.000, Figure 5). Subgroup analysis based on different subtypes of HF revealed a noteworthy reduction in TNF-α expression levels for patients with HFrEF (MD = −8.11, 95% CI: −10.50 to −5.72, *p* = 0.000, Figure 5) and HFmrEF (MD = −7.63, 95% CI: −9.86 to −6.00, *p* = 0.000, Figure 5) when the combination therapy was administered. Interestingly, the reduction was particularly prominent among patients with HFrEF.

#### 3.4.2 IL-6

**FIGURE 6:**
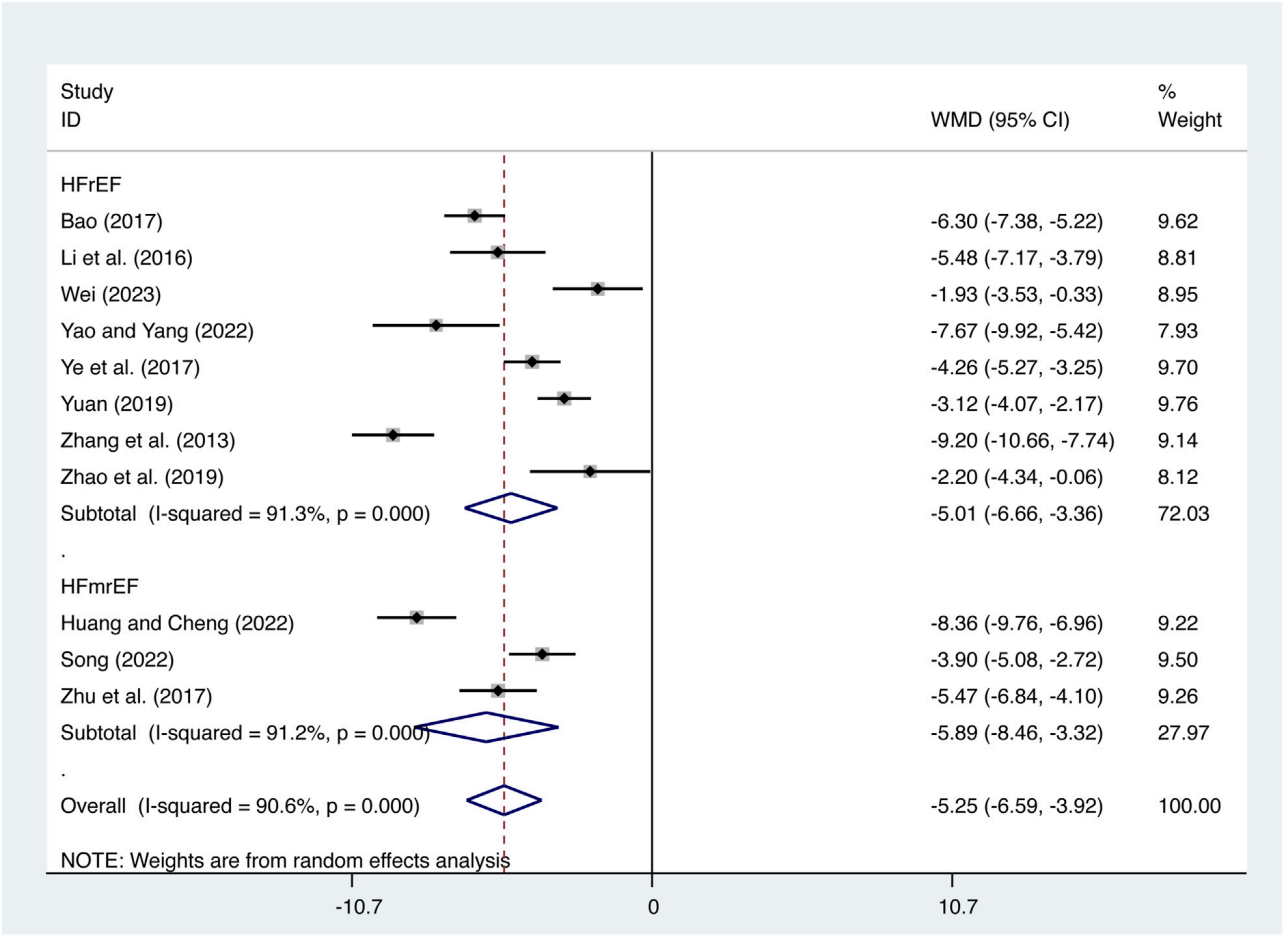
Forest plot for IL-6 expression levels.

11 studies (Bao, 2017; Huang and Cheng, 2022; T. T; Li et al., 2016; Song, 2022; Wei, 2023; Yao and Yang, 2022; Ye et al., 2017; Yuan, 2019; Zhang et al., 2013; Zhao et al., 2019; Zhu et al., 2017) evaluated IL-6 expression levels with high heterogeneity (*I* ^*2*^ = 90.6%, *p* = 0.000) and merged it with a random-effects model. The combination therapy of XMLI and CT significantly reduced IL-6 expression levels compared to CT alone (MD = −5.25, 95% CI: −6.59 to −3.92, *p* = 0.000, Figure 6). Subgroup analysis based on different subtypes of HF revealed a noteworthy reduction in IL-6 expression levels for patients with HFrEF (MD = −5.01, 95% CI: −6.66 to −3.36, *p* = 0.000, Figure 6) and HFmrEF (MD = −5.89, 95% CI: −8.46 to −3.32, *p* = 0.000, Figure 6) when the combination therapy was administered. Interestingly, the reduction was particularly prominent among patients with HFmrEF.

#### 3.4.3 IL-10

**FIGURE 7:**
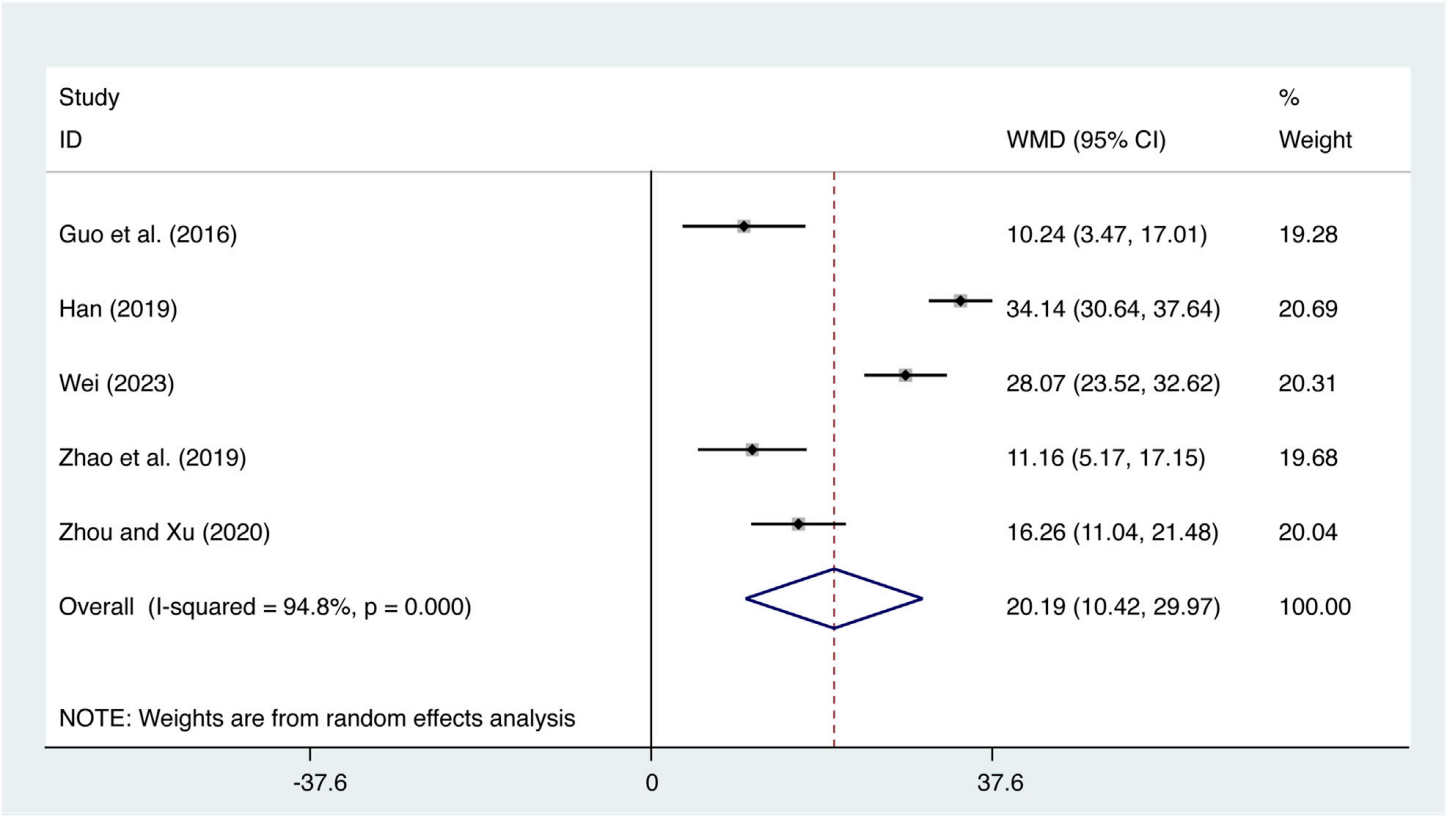
Forest plot for IL-10 expression levels.

Five studies (Guo et al., 2016; Han, 2019; Zhao et al., 2019; Zhou and Xu, 2020; Wei, 2023) evaluated IL-10 expression levels with high heterogeneity (*I* ^*2*^ = 94.8%, *p* = 0.000) and merged it with a random-effects model. The combination therapy of XMLI and CT significantly increased IL-10 expression levels compared to CT alone (MD = 20.19, 95% CI: 10.42 to 29.97, *p* = 0.000, Figure 7).

#### 3.4.4 IL-18

**FIGURE 8:**
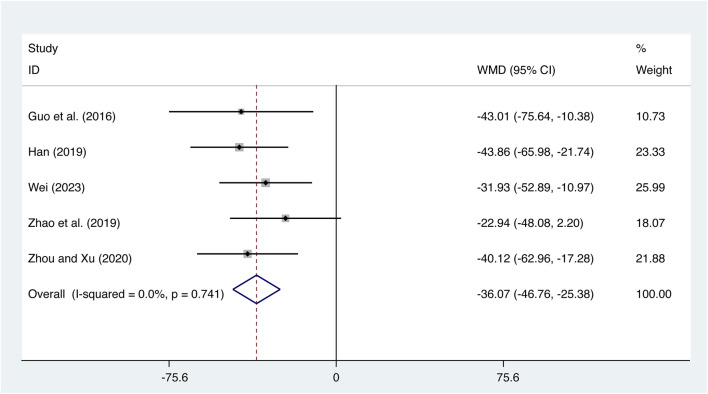
Forest plot for IL-18 expression levels.

Five studies (Guo et al., 2016; Han, 2019; Zhao et al., 2019; Zhou and Xu, 2020; Wei, 2023) evaluated IL-18 expression levels with low heterogeneity (*I* ^*2*^ = 0.0%, *p* = 0.741) and merged it with a fixed-effects model. The combination therapy of XMLI and CT significantly reduced IL-18 expression levels compared to CT alone (MD = −36.07, 95% CI: −46.76 to −25.38, *p* = 0.000, Figure 8).

#### 3.4.5 CRP

**FIGURE 9:**
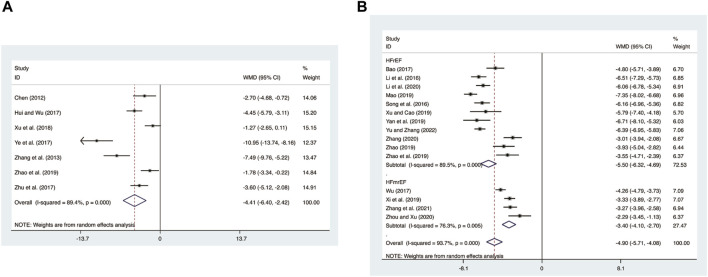
Forest plot for CRP and hs-CRP expression levels. **(A)** CRP. **(B)** hs-CRP.

Seven studies (Chen, 2012; Zhang et al., 2013; Hui and Wu, 2017; Ye et al., 2017; Zhu et al., 2017; Xu et al., 2018; Zhao, 2019) evaluated CRP expression levels with high heterogeneity (*I* ^*2*^ = 89.4%, *p* = 0.000) and merged it with a random-effects model. The combination therapy of XMLI and CT significantly reduced CRP expression levels compared to CT alone (MD = −4.41, 95% CI: −6.40 to −2.42, *p* = 0.000, Figure 9A).

#### 3.4.6 hs-CRP

15 studies (Bao, 2017; T. T; Li et al., 2016; Li et al., 2020; Mao, 2019; Song et al., 2016; Wu et al., 2017; Xi et al., 2019; Xu and Cao, 2019; Yan et al., 2019; Yu and Zhang, 2023; Zhang et al., 2021; Zhang, 2020; Zhao et al., 2019; Zhao, 2019; Zhou and Xu, 2020) evaluated hs-CRP expression levels with high heterogeneity (*I* ^*2*^ = 93.7%, *p* = 0.000) and merged it with a random-effects model. The combination therapy of XMLI and CT significantly reduced hs-CRP expression levels compared to CT alone (MD = −4.90, 95% CI: −5.71 to −4.08, *p* = 0.000, Figure 9B). Subgroup analysis based on different subtypes of HF revealed a noteworthy reduction in hs-CRP expression levels for patients with HFrEF (MD = −5.50, 95% CI: −6.32 to −4.69, *p* = 0.000, Figure 9B) and HFmrEF (MD = −3.40, 95% CI: −4.10 to −2.70, *p* = 0.000, Figure 9B) when the combination therapy was administered. Interestingly, the reduction was particularly prominent among patients with HFrEF.

### 3.5 Secondary outcomes

#### 3.5.1 Clinical efficacy

**FIGURE 10:**
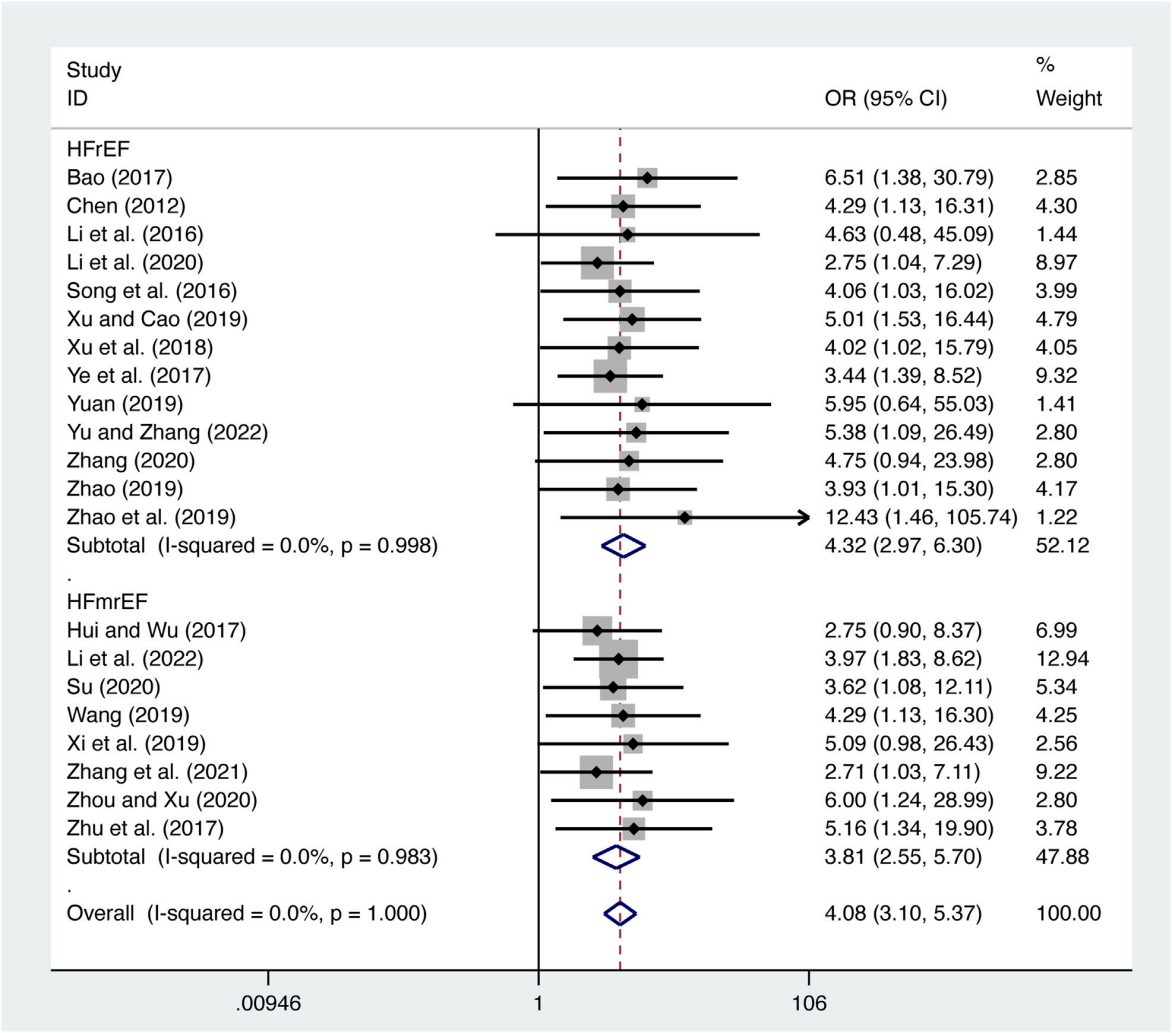
Forest plot for clinical efficacy.

21 studies (Bao, 2017; Chen, 2012; Hui and Wu, 2017; Li et al., 2022; T. T; Li et al., 2016; Li et al., 2020; Song et al., 2016; Su, 2020; Wang, 2019; Xi et al., 2019; Xu et al., 2018; Xu and Cao, 2019; Ye et al., 2017; Yu and Zhang, 2023; Yuan, 2019; Zhang et al., 2021; Zhang, 2020; Zhao et al., 2019; Zhao, 2019; Zhou and Xu, 2020; Zhu et al., 2017) evaluated clinical efficacy with low heterogeneity (*I* ^*2*^ = 0.0%, *p* = 1.000) and merged it with a fixed-effects model. The combination therapy of XMLI and CT significantly improved clinical efficacy compared to CT alone (OR = 4.08, 95% CI: 3.10 to 5.37, *p* = 0.000, Figure 10). Subgroup analysis based on different subtypes of HF revealed a noteworthy enhancement in clinical efficacy for patients with HFrEF (OR = 4.32, 95% CI: 2.97 to 6.30, *p* = 0.000, Figure 10) and HFmrEF (OR = 3.81, 95% CI: 2.55 to 5.70, *p* = 0.000, Figure 10) when the combination therapy was administered. Interestingly, the improvement was particularly prominent among patients with HFrEF.

#### 3.5.2 6-MWD

**FIGURE 11:**
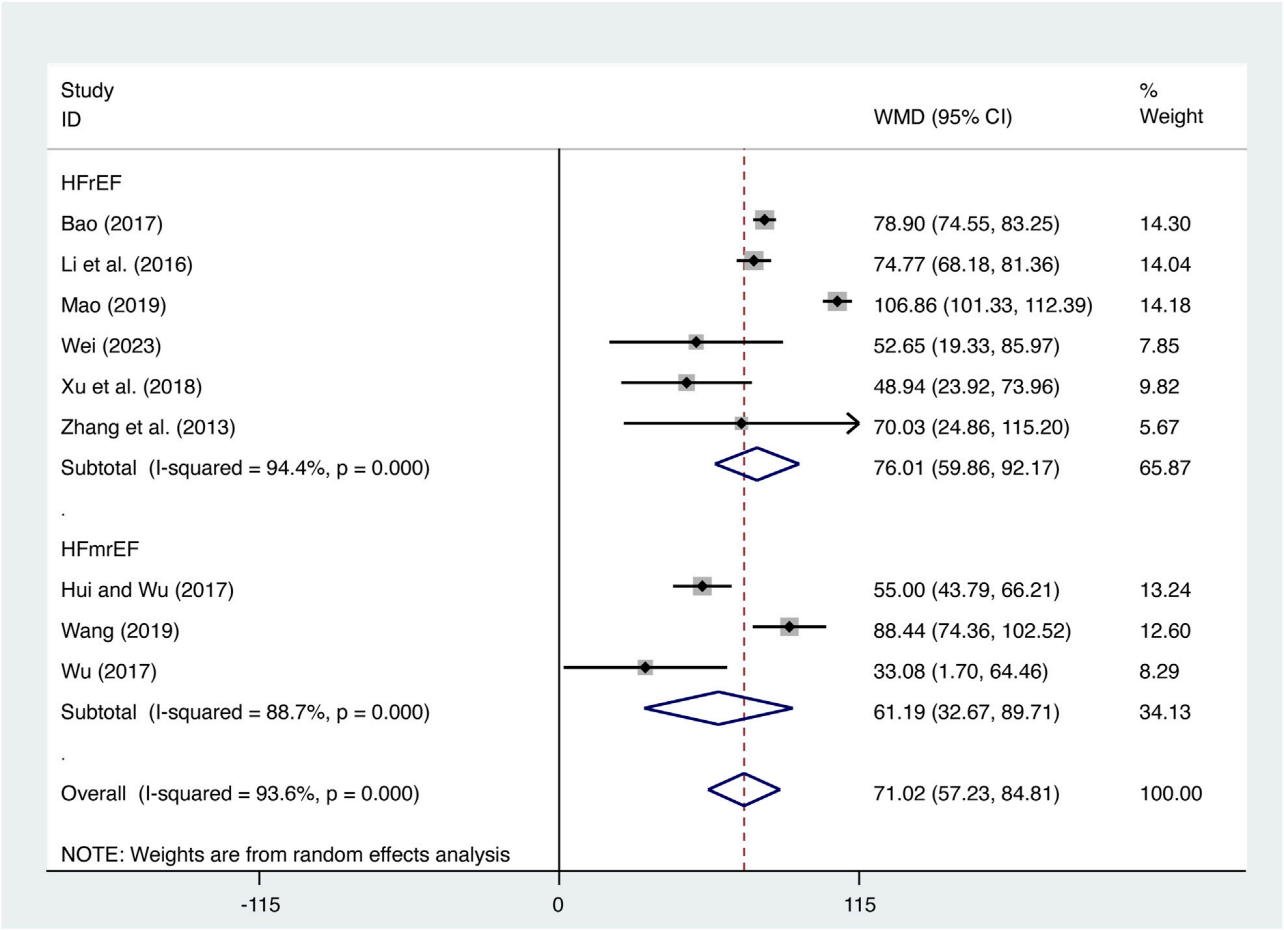
Forest plot for 6-MWD.

Nine studies (Bao, 2017; Hui and Wu, 2017; T. T; Li et al., 2016; Mao, 2019; Wang, 2019; Wei, 2023; Wu et al., 2017; Xu et al., 2018; Zhang et al., 2013) evaluated 6-MWD with high heterogeneity (*I* ^*2*^ = 93.6%, *p* = 0.000) and merged it with a random-effects model. The combination therapy of XMLI and CT significantly increased 6-MWD compared to CT alone (MD = 71.02, 95% CI: 57.23 to 84.81, *p* = 0.000, Figure 11). Subgroup analysis based on different subtypes of HF revealed a noteworthy enhancement in 6-MWD for patients with HFrEF (MD = 76.01, 95% CI: 59.86 to 92.17, *p* = 0.000, Figure 11) and HFmrEF (MD = 61.19, 95% CI: 32.67 to 89.71, *p* = 0.000, Figure 11) when the combination therapy was administered. Interestingly, the improvement was particularly prominent among patients with HFrEF.

#### 3.5.3 BNP

**FIGURE 12:**
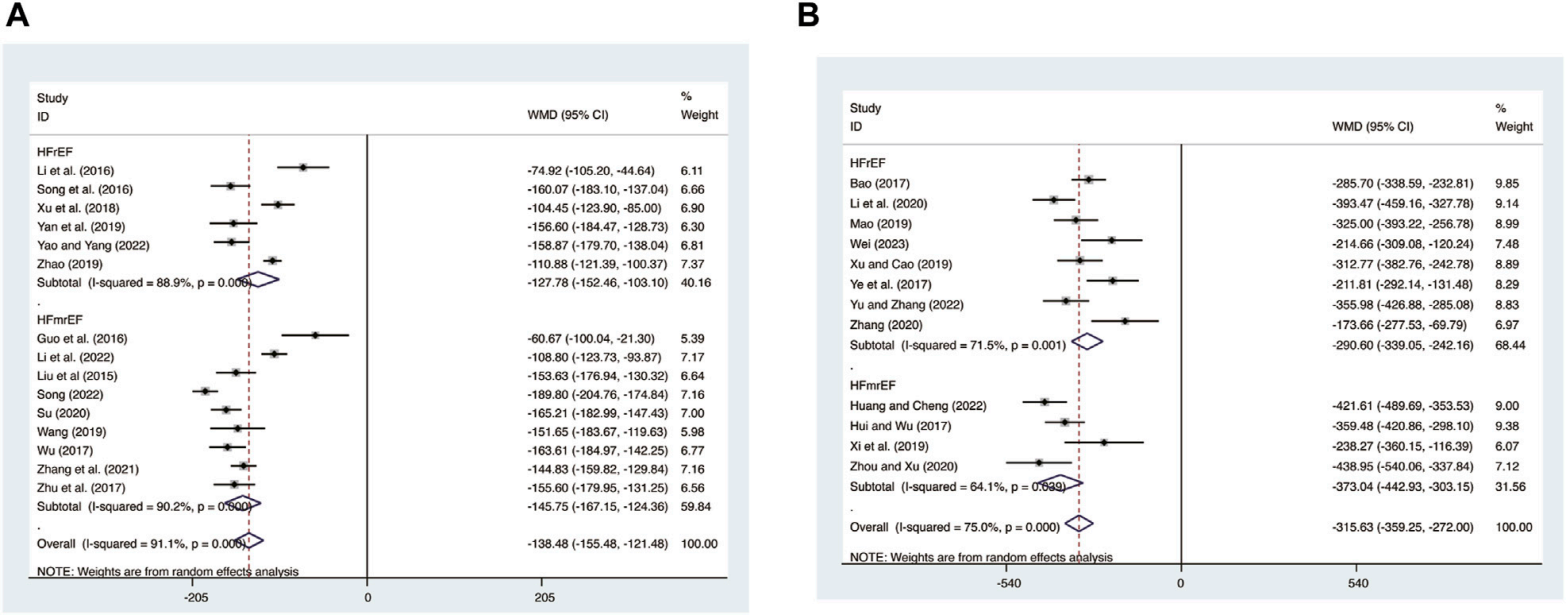
Forest plot for BNP and NT-pro BNP expression levels. **(A)** BNP. **(B)** NT-pro BNP.

15 studies (Guo et al., 2016; Li et al., 2022; T. T; Li et al., 2016; Liu et al., 2015; Song, 2022; Song et al., 2016; Su, 2020; Wang, 2019; Wu et al., 2017; Xu et al., 2018; Yan et al., 2019; Yao and Yang, 2022; Zhang et al., 2021; Zhao, 2019; Zhu et al., 2017) evaluated BNP expression levels with high heterogeneity (*I* ^*2*^ = 91.1%, *p* = 0.000) and merged it with a random-effects model. The combination therapy of XMLI and CT significantly reduced BNP expression levels compared to CT alone (MD = −138.48, 95% CI: −155.48 to −121.48, *p* = 0.000, Figure 12A). Subgroup analysis based on different subtypes of HF revealed a noteworthy reduction in BNP expression levels for patients with HFrEF (MD = −127.78, 95% CI: −152.46 to −103.10, *p* = 0.000, Figure 12A) and HFmrEF (MD = −145.75, 95% CI: −167.15 to −124.36, *p* = 0.000, Figure 12A) when the combination therapy was administered. Interestingly, the reduction was particularly prominent among patients with HFmrEF.

#### 3.5.4 NT-pro BNP

12 studies (Bao, 2017; Hui and Wu, 2017; Ye et al., 2017; Mao, 2019; Xi et al., 2019; Xu and Cao, 2019; Li et al., 2020; Zhang, 2020; Zhou and Xu, 2020; Huang and Cheng, 2022; Wei, 2023; Yu and Zhang, 2023) evaluated NT-pro BNP expression levels with high heterogeneity (*I* ^*2*^ = 75.0%, *p* = 0.000) and merged it with a random-effects model. The combination therapy of XMLI and CT significantly reduced NT-pro BNP expression levels compared to CT alone (MD = −315.63, 95% CI: −359.25 to −272.00, *p* = 0.000, Figure 12B). Subgroup analysis based on different subtypes of HF revealed a noteworthy reduction in NT-pro BNP expression levels for patients with HFrEF (MD = −290.60, 95% CI: −339.05 to −242.16, *p* = 0.000, Figure 12B) and HFmrEF (MD = −373.04, 95% CI: −442.93 to −303.15, *p* = 0.000, Figure 12B) when the combination therapy was administered. Interestingly, the reduction was particularly prominent among patients with HFmrEF.

#### 3.5.5 Adverse reactions

**FIGURE 13:**
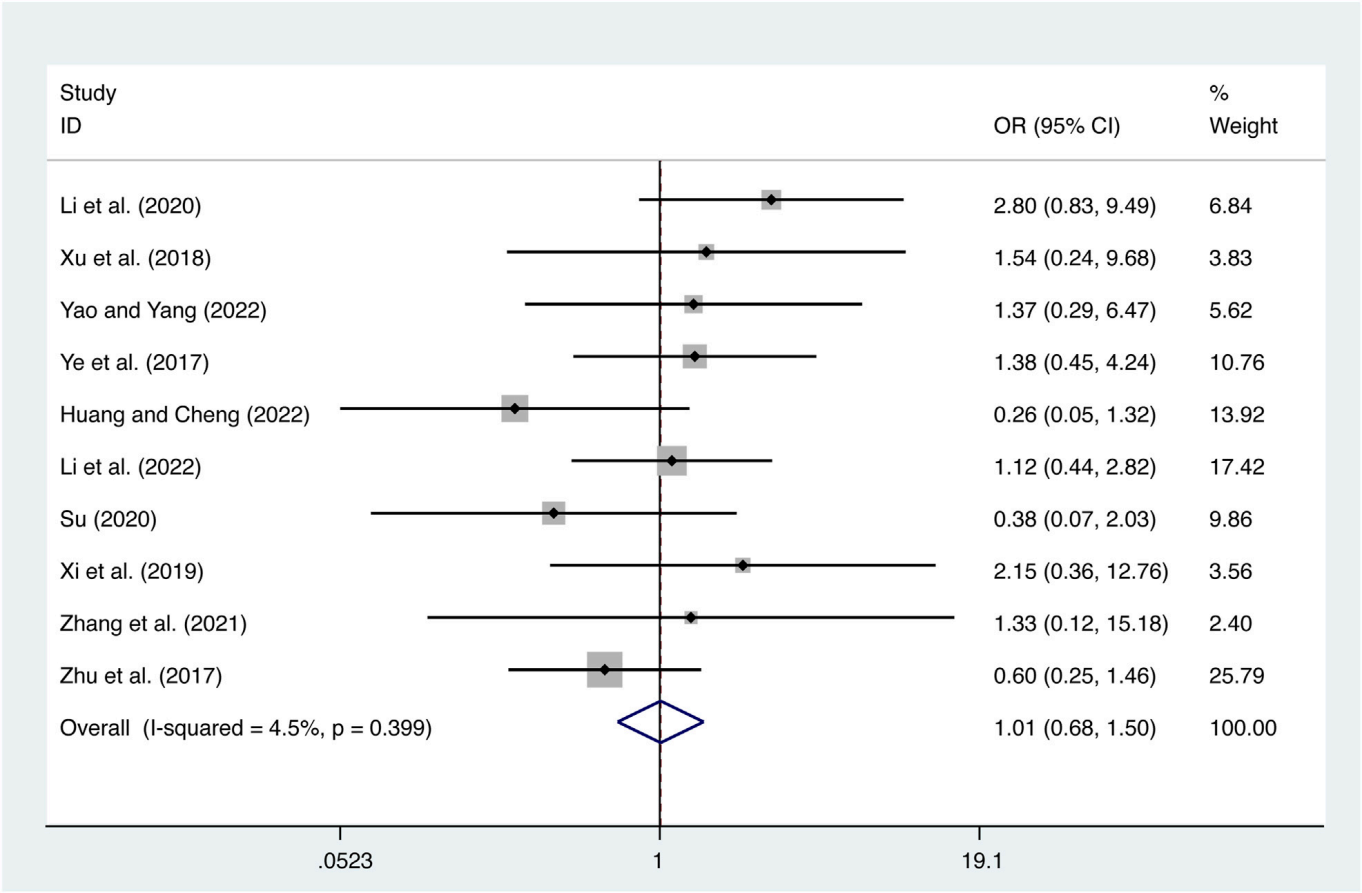
Forest plot for adverse reactions.

10 studies (Ye et al., 2017; Zhu et al., 2017; Xu et al., 2018; Xi et al., 2019; Li et al., 2020; Su, 2020; Zhang et al., 2021; Huang and Cheng, 2022; Li et al., 2022; Yao and Yang, 2022) reported on adverse reactions with low heterogeneity (*I* ^*2*^ = 4.5%, *p* = 0.399) and merged it with a fixed-effects model. The combination therapy of XMLI and CT revealed no significant difference in adverse reactions compared to CT alone (OR = 1.01, 95% CI: 0.68 to 1.50, *p* = 0.97, Figure 13). The study drug is associated with several common adverse reactions, including dizziness, headache, nausea, vomiting, diarrhea, palpitations, fatigue, rash, hypokalemia, dyspnea, hypotension, tachycardia, and liver dysfunction. It is important to note that these adverse reactions typically subside with appropriate symptomatic treatment. Notably, none of the participants in the study discontinued the use of the drug as a result of experiencing adverse reactions. For more comprehensive information, please consult the Supplementary Material.

### 3.6 Sensitivity analysis

**FIGURE 14:**
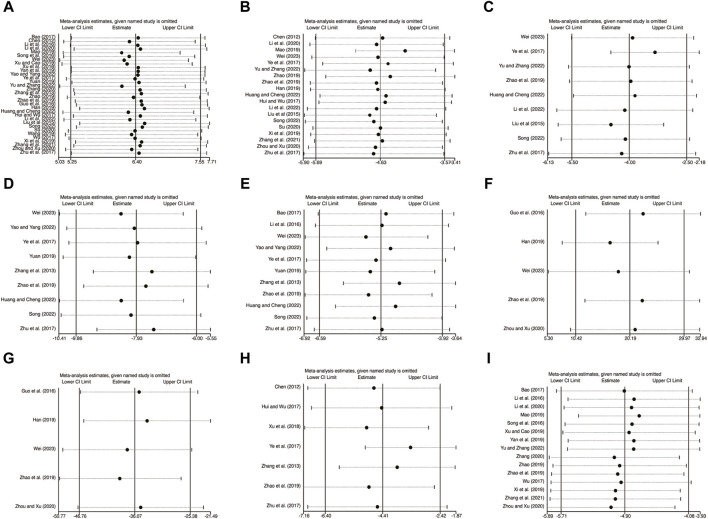
The results of sensitivity analysis. **(A)** LVEF. **(B)** LVEDD. **(C)** LVESD. **(D)** TNF-α. **(E)** IL-6. **(F)** IL-10. **(G)** IL-18. **(H)** CRP. **(I)** hs-CRP.

To assess the reliability and robustness of the consolidation results, a sensitivity analysis was conducted. This analysis involved sequentially excluding individual studies and examining their impact on various variables, including LVEF (Figure 14A), LVEDD (Figure 14B), LVESD (Figure 14C), TNF-α (Figure 14D), IL-6 (Figure 14E), IL-10 (Figure 14F), IL-18 (Figure 14G), CRP (Figure 14H), and hs-CRP (Figure 14I). Interestingly, the exclusion of any of these studies had no significant effect on the combined results. This finding suggests that the merged results are both robust and reliable, as clearly shown in Figure 14.

### 3.7 Publication bias

**FIGURE 15:**
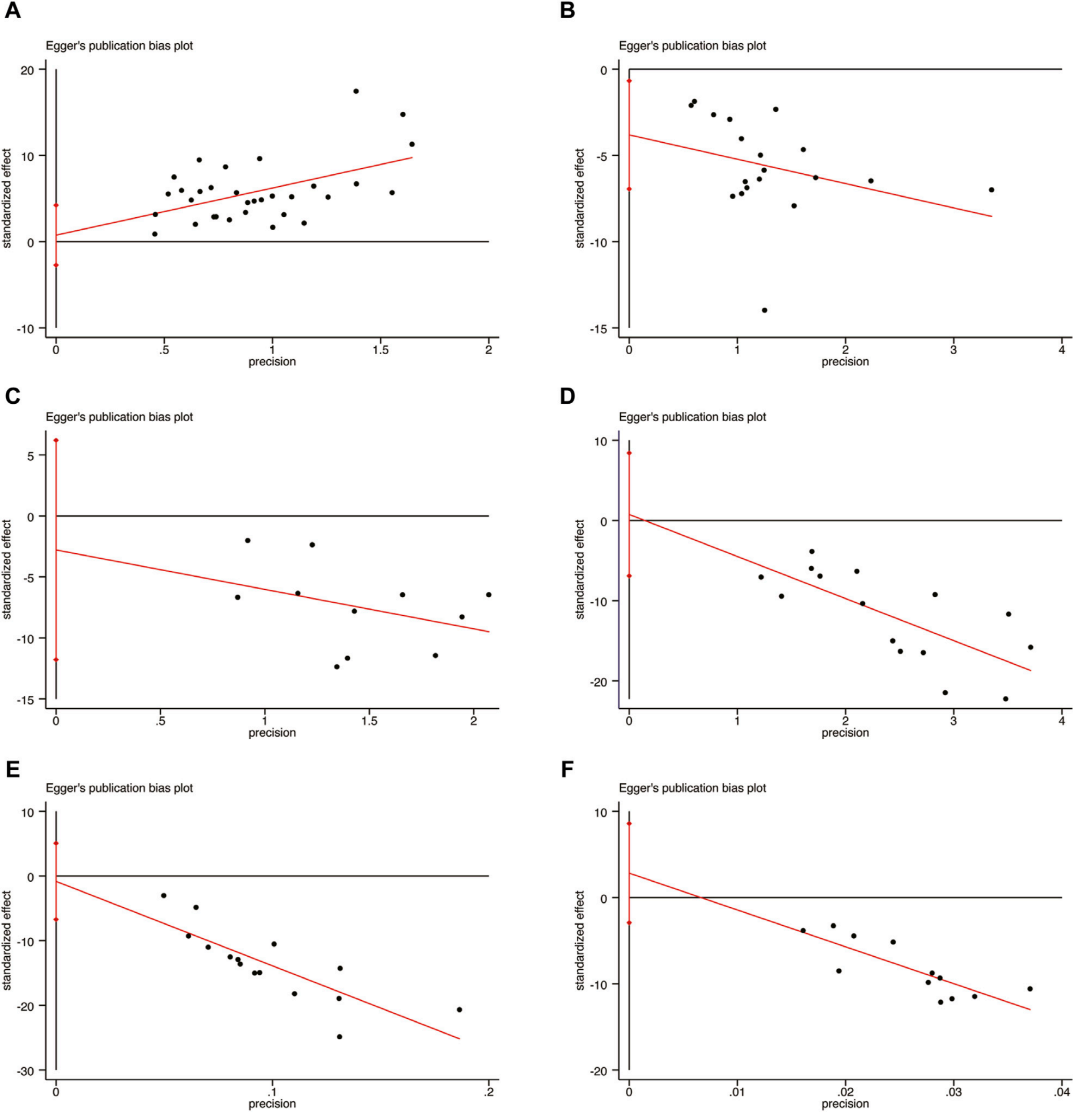
Egger’s publication funnel plot. **(A)** LVEF. **(B)** LVEDD. **(C)** IL-6. **(D)** hs-CRP. **(E)** BNP. **(F)** NT-pro BNP.

In order to evaluate publication bias, the Egger’s test was utilized specifically for LVEF, LVEDD, IL-6, hs-CRP, BNP, and NT-pro BNP, as depicted in Figure 15. Remarkably, the results of the analysis revealed that there was no significant publication bias for LVEF (Figure 15A, *p* = 0.667), LVEDD (Figure 15B, *p* = 0.188), IL-6 (Figure 15C, *p* = 0.500), hs-CRP (Figure 15D, *p* = 0.836), BNP (Figure 15E, *p* = 0.767), and NT-pro BNP (Figure 15F, *p* = 0.298).

## 4 Discussion

### 4.1 Summary of findings

This meta-analysis is the first to investigate the effects of XMLI on LVR and inflammatory mediators in patients with CHF. A total of 32 RCTs were included in this analysis, revealing several important findings. Firstly, the combination of XMLI and CT significantly improved LVR in HF patients. This improvement was supported by an increase in LVEF, as well as a decrease in LVEDD and LVESD. As well as levels of BNP, and NT-pro BNP were decreased. Furthermore, the combination therapy also resulted in a significant reduction in inflammatory mediators. Specifically, there was a decrease in the expression levels of pro-inflammatory cytokines TNF-a, IL-6, IL-18, CRP, and hs-CRP. Conversely, there was an increase in the expression levels of the anti-inflammatory cytokine IL-10. In addition to improving LVR and reducing inflammatory mediators, the combination therapy showed higher clinical efficacy and improvement of the 6-MWD. Importantly, the combination therapy demonstrated good safety, with only minor adverse events reported. These events were manageable in terms of symptoms and had no impact on treatment outcomes. Based on these compelling results, our meta-analysis suggests that XMLI effectively improves LVR in HF patients, reduces inflammatory mediators, and enhances overall clinical efficacy.

To ensure the robustness and reliability of our findings, sensitivity analysis was conducted. Individual studies were sequentially deleted, and sensitivity analysis was performed on key indicators of LVR and inflammatory mediators, further confirming the validity of our results. Additionally, Egger’s test was conducted to evaluate publication bias, with the results showing no significant publication bias. This further strengthens the validity and reliability of our findings.

### 4.2 Comparison with previous studies

Although Lu et al.'s (Lu et al., 2018) previous meta-analysis evaluated the clinical efficacy of XMLI in treating CHF, our study specifically focuses on its effects on LVR and inflammatory mediators. It should be noted that there are several shortcomings in previous studies: Firstly, HF is classified into different subtypes based on ejection fraction according to the HF management guidelines. However, previous studies did not consider these subtypes or conduct subgroup analysis based on ejection fraction during meta-analysis, which may introduce heterogeneity and bias. Secondly, sensitivity analysis was not performed, and only funnel plots were used to evaluate publication bias in previous studies, which affects the robustness and reliability of the research findings. Finally, previous studies mainly focused on clinical indicators such as symptom improvement, exercise tolerance, and quality of life in CHF patients treated with XMLI. The impact of XMLI on LVR and inflammatory mediators has not been extensively explored. Understanding these specific effects is crucial for a comprehensive assessment of XMLI’s therapeutic potential in CHF patients.

### 4.3 Strengths and limitations

This meta-analysis of RCTs is the first to specifically investigate the effects of XMLI on LVR and inflammatory mediators in patients with CHF. To enhance the reliability of our findings, we will conduct subgroup analyses based on different types of HF, eliminating potential confounding factors associated with disease types. Additionally, our study addresses a crucial aspect of CHF by evaluating the impact of XMLI on LVR, a key pathological and physiological mechanism contributing to high hospitalization and mortality rates in CHF patients. Furthermore, we comprehensively evaluate the role of inflammatory response in LVR, highlighting its significance in the progression of HF, an aspect that previous studies have yet to fully address.

Several limitations should be considered when interpreting the results of this study. Firstly, the included studies in this meta-analysis exhibit relatively low overall quality, with limited reporting of allocation concealment and blinding, which poses a serious risk of bias. Secondly, significant heterogeneity is observed among the RCTs, although subgroup and sensitivity analyses are conducted without identifying the sources of heterogeneity. This variation may be associated with the lack of standardized dosage and intervention duration. Thirdly, the small size of the included studies highlights the need for larger scale research to ensure result reliability. Therefore, caution should be exercised when interpreting the findings. Fourthly, it is important to note that all studies included in this analysis were conducted in China and exclusively involved Chinese participants. This limited geographical scope may introduce sources of heterogeneity. To ensure the applicability of these findings to different races, future studies should incorporate with more diverse samples from various geographical regions. Lastly, the limited number of studies examining TNF-α, IL-10, IL-8, and CRP results in low supporting evidence. Future research should prioritize larger scale and more rigorous studies to verify the stability of our findings.

### 4.4 Implication

To strengthen the evidence regarding the efficacy of XMLI treatment for CHF, future clinical research should address the following areas. Firstly, studies should be conducted on different types of HF to comprehensively assess the efficacy of XMLI in treating HF. By doing so, bias can be minimized, and more accurate conclusions can be generated. Secondly, rigorous adherence to clinical research standards, including strict randomization, allocation concealment, and blinding techniques, should be ensured. Encouraging placebo-controlled randomized trials can provide more precise results. Thirdly, it is crucial to report RCTs in a complete and comprehensive manner by employing standardized reporting trial statements. Alongside primary outcome measures, additional information such as comorbidities, disease duration, medication usage, readmission rates, follow-up duration, and endpoint time should be reported, facilitating the analysis of heterogeneity and prognosis clarification. Finally, given the significant role of LVR and inflammation in the progression of HF, future research should focus on targeted and high-quality studies in these areas.

## 5 Conclusion

The results of the systematic review and meta-analysis suggest that the combination of XML and CT can effectively improve LVR and reduce inflammatory mediators in CHF patients, with a good safety profile. However, it is crucial to approach these findings with caution due to the low level of evidence and high heterogeneity observed in the included studies, particularly in regard to the evaluation of inflammatory mediators. To strengthen these conclusions, future research should prioritize high-quality RCTs that can provide more substantive evidence.

## Data Availability

The original contributions presented in the study are included in the article/Supplementary Material, further inquiries can be directed to the corresponding authors.
