# Supplementary material for: Xinmailong injection on left ventricular remodeling and inflammatory mediators in patients with CHF: a systematic review and meta-analysis
Source: Front Pharmacol. 2024 Apr 9;15:1370448. doi: 10.3389/fphar.2024.1370448 (PMC11035828; doi:10.3389/fphar.2024.1370448)
Supplement: Supplementary file 1 [file Table1.DOCX]

Supplementary Material

**Effect of Xinmailong injection on left ventricular remodeling and inflammatory mediators in patients with CHF: a systematic review and meta-analysis**

Xu Han^1^, Xi Chen^1^, Yanling Liu^2^, Jie Yang^2^, Wuzhi Nie^2^, Mingjiu Yang^2^* and Xinglang Mou^2^*

^1^Chongqing Changshou Traditional Chinese Medicine Hospital, Chongqing, China, ^2^ Traditional Chinese Medicine Hospital Dianjiang Chongqing, Chongqing, China

*Correspondence: Mingjiu Yang, 13896346371@163.com; Xinglang Mou, djxzyymxl@163.com. Traditional Chinese Medicine Hospital Dianjiang Chongqing, Chongqing, 408300, China.

# The search strategy

**Search run on December 31 2023**

**PubMed (*n*=23)**

#1 ((xinmailong injection[Title/Abstract]) OR (xinmailong[Title/Abstract]))

#2 ((heart failure[Title/Abstract]) OR (chronic heart failure[Title/Abstract]))

#3 #1 AND #2

**Embase (*n*=17)**

#1 'xinmailong injection'/exp

#2 'xinmailong'/exp

#3 #1 OR #2

#4 'heart failure'/exp

#5 'chronic heart failure'/exp

#6 #4 OR #5

#7 #3 AND #6

**Cochrane library (*n*=4)**

#1 MeSH descriptor: [xinmailong injection] explode all trees

#2 xinmailong injection* or xinmailong*.ti,ab,kw

#3 #1 or #2

#4 MeSH descriptor: [heart failure] explode all trees

#5 chronic heart failure*.ti,ab,kw

#6 #4 or #5

#7 #3 and #6

**Web of Science (*n*=19)**

#1 (TS=(xinmailong injection) OR ALL=(xinmailong))

#2 (TS=(heart failure) OR ALL=(chronic heart failure))

#3 #1 AND #2

**CNKI (*n*=341)**

#1 篇关摘：心脉隆 OR 心脉隆注射液

#2 篇关摘：心力衰竭 OR 慢性心力衰竭

#3 #1 AND #2

**Wanfang Data (*n*=363)**

#1 主题：心脉隆 OR 心脉隆注射液

#2 主题：心力衰竭 OR 慢性心力衰竭

#3 #1 AND #2

**VIP (*n*=298)**

#1 篇关摘：心脉隆 OR 心脉隆注射液

#2 篇关摘：心力衰竭 OR 慢性心力衰竭

#3 #1 AND #2

**CBM (*n*=337)**

#1 常用字段：心脉隆 OR 心脉隆注射液

#2 常用字段：心力衰竭 OR 慢性心力衰竭

#3 #1 AND #2

# Supplementary table

**Supplementary table 1 The incidence rate of adverse reactions.**

| Adverse reaction symptoms | First author  (publication year) | The number of adverse reactions | |
| --- | --- | --- | --- |
|  |  | T | C |
| Dizziness | Song et al. (2016); Song et al. (2016); Su (2020); Yao and Yang (2022); Zhu et al. (2017) | 7 | 8 |
| Headache | Li et al. (2022); Xu et al. (2018); Ye et al. (2017); Ye et al. (2017); Zhu et al. (2017) | 8 | 6 |
| Nausea | Huang and Cheng (2022); Li et al. (2020); Li et al. (2022); Song et al. (2016); Song et al. (2016); Su (2020); Xi et al. (2019); Xu et al. (2018); Yao and Yang (2022); Zhang et al. (2021); Zhu et al. (2017) | 13 | 11 |
| Vomiting | Li et al. (2020) Li et al. (2022); Ye et al. (2017) | 4 | 7 |
| Diarrhea | Huang and Cheng (2022); Yao and Yang (2022) | 2 | 3 |
| Palpitations | Li et al. (2022); Song et al. (2016); Song et al. (2016); Su (2020); Xi et al. (2019); Ye et al. (2017) | 6 | 9 |
| Fatigue | Li et al. (2020); Su (2020); Xi et al. (2019) | 3 | 2 |
| Rash | Huang and Cheng (2022) | 0 | 2 |
| Hypokalemia | Ye et al. (2017) | 1 | 0 |
| Dyspnea | Xi et al. (2019) | 1 | 0 |
| Hypotension | Xu et al. (2018); Zhang et al. (2021); Zhu et al. (2017) | 6 | 5 |
| Tachycardia | Xu et al. (2018); Zhang et al. (2021); Zhu et al. (2017) | 4 | 2 |
| Liver dysfunction | Ye et al. (2017) | 3 | 2 |
| Total reactions | － | 58/640 | 57/618 |
| Incidence rate | － | 9.06% | 9.22% |
